# Aeromedical interhospital transport of an adult with COVID-19 on extracorporeal membrane oxygenation: case report

**DOI:** 10.1590/1980-220X-REEUSP-2021-0432

**Published:** 2022-03-14

**Authors:** Vânia Paula de Carvalho, Bruno Gonçalves da Silva, Flávio Lopes Ferreira, André Alves Elias, Armando Sergio de Aguiar, Nelson Miguel Galindo

**Affiliations:** 1 Universidade Federal de Minas Gerais, Belo Horizonte, MG, Brazil.; 2 Universidade FUMEC, Belo Horizonte, MG, Brazil.; 3 Universidade José do Rosário Vellano, Belo Horizonte, MG, Brazil.; 4 Instituto Federal de Educação, Ciência e Tecnologia de Pernambuco, Pesqueira, PE, Brazil.

**Keywords:** Coronavirus Infections, Extracorporeal membrane oxygenation, Inter-hospital transport, Knowledge management, Case Reports, Infecciones por coronavirus, Oxigenación por membrana extracorpórea, Transporte interhospitalario, Conocimiento administrativo, Reportes del caso, Infecções por Coronavírus, Oxigenação por Membrana Extracorpórea, Transporte Inter-hospitalar, Gestão do Conhecimento, Relatos de Casos

## Abstract

**Objective:**

To describe the experience of aeromedical interhospital transport of an adult patient with severe hypoxemic respiratory failure due to SARS-CoV-2, on extracorporeal membrane oxygenation.

**Method:**

This is a case report, guided by the tool Case Report Guidelines, with a descriptive approach. Data were collected from the digital medical record and field notes after the approval by the Institution and the Human Research Ethics Committee.

**Results:**

The transport of a critically ill, unstable patient with acute respiratory syndrome 2 on extracorporeal oxygenation was an opportunity for the team to acquire new knowledge. The proper preparation of the fixed-wing aircraft and the profile of the team of specialist nurses contributed to the safety and quality in the three phases of flight: preflight, in-flight and post-flight.

**Conclusion:**

Air transport of adults on cardiopulmonary bypass to referral centers, under the care of an experienced multidisciplinary team, can contribute to positive results. The nurses’ autonomy, their leadership role and expertise in process management are highlighted. Thus, success was evidenced with the patient’s discharge after 45 days from the Intensive Care Unit.

## RESUMO

**Objetivo:**

Descrever a experiência do transporte inter-hospitalar aeromédico de um paciente adulto, com insuficiência respiratória hipoxêmica grave por SARS-CoV-2, em uso de membrana de oxigenação extracorpórea.

**Método:**

Relato de caso, norteado pela ferramenta Case Report Guidelines, com abordagem descritiva. Os dados foram coletados do prontuário digital e do diário de campo após aprovação feita pela Instituição e pelo Comitê de Ética e Pesquisa com Seres Humanos.

**Resultados:**

O transporte do paciente em estado crítico, instável, com síndrome respiratória aguda 2 em uso de oxigenação extracorpórea foi uma oportunidade para a equipe assimilar novos conhecimentos. O preparo adequado da aeronave de asa fixa e o perfil da equipe de enfermeiros especialistas contribuíram para realizar com segurança e qualidade as três fases de voo: pré-voo, durante o voo e pós voo.

**Conclusão:**

O transporte aéreo de adultos para os centros de referência em circulação extracorpórea, sob os cuidados de uma equipe multiprofissional experiente, pode contribuir para os resultados positivos. Destaca-se a autonomia dos enfermeiros, o papel de liderança e a expertise no gerenciamento de processos. Para tal, o sucesso foi evidenciado pela alta do paciente após 45 dias do Centro de Terapia Intensiva.

## RESUMEN

**Objetivo:**

Describir la experiencia del transporte inter hospitalario aeromédico de un paciente adulto, con hipoxia grave por SARS-CoV-2, en uso de membrana de oxigenación extra corpórea.

**Método:**

Reporte de caso, guiado por la herramienta *Case Report Guidelines*, con abordaje descriptivo. Los datos fueron recolectados del historial clínico digital del paciente y de los apuntes de campo tras aprobación hecha por la Institución y por el Comité de Ética e Investigación con Seres Humanos.

**Resultados:**

El transporte del paciente en estado grave, instable, con síndrome respiratoria aguda 2 en uso de oxigenación extra corpórea fue una oportunidad para el equipo asimilar nuevos conocimientos. La preparación adecuada de la aeronave de ala fija y el perfil del equipo de enfermeros expertos contribuyeron para realizar con seguridad y calidad las tres etapas del vuelo: antes, durante y después del vuelo.

**Conclusión:**

El transporte aéreo de adultos a los centros de referencia en circulación extra corpórea, bajo los cuidados de un equipo con diversos profesionales experientes, puede ser contributivo para los resultados positivos. Se pone de relieve la autonomía de los enfermeros, el rol de liderazgo y la *expertise* en el manejo de procesos. Para tal, el éxito fue evidenciado por el alta al paciente después de 45 días en Unidad de Cuidados Intensivos.

## INTRODUCTION

Coronavirus Disease 2019 (COVID-19) has changed the world health scenario and interhospital aeromedical transport^(1)^. In this scenario, planned care, effective communication, and teamwork proved to be essential for the safety of everyone involved in this type of transport^(2–3)^. Therefore, as a result of the COVID-19 pandemic, frontline health workers had to face the dangers to which they are exposed, seek innovative solutions, and share information of successful experiences.

It is understood that aeromedical transport involves a complex and specific logistics service provided by professionals who work based on advanced practices^(4–5)^. As a means considered fast and effective, it has been increasingly used by public and private health systems worldwide^(3)^, especially for severe cases requiring rapid transfer to referral hospitals.

Among the cases of high severity, there is severe acute respiratory syndrome 2 (SARS-CoV-2), caused by the Coronavirus, which can lead to severe respiratory deterioration, and Acute Respiratory Distress Syndrome (ARDS), which will require intensive care from highly complex services.

For transport to be effective, several therapeutic strategies can be used to maintain life. Backed by the guidelines of the Extracorporeal Life Support Organization (ELSO), for the treatment and vital maintenance of cases of severe respiratory impairment, the use of extracorporeal membrane oxygenation (ECMO) is a relevant option in the face of severe pulmonary impairment, since this process allows the lung to continue to perform its attribution of gas exchange, with consequent blood oxygenation^(6)^.

In Brazil, there are 13 centers accredited by ELSO, 54% located in the southeastern region of the country; however, there are no guidelines for interhospital transfer of ECMO-eligible patients to reference centers^(7)^.

ECMO is a temporary, mechanical support system responsible for controlling the cardiopulmonary function of critically ill patients with a variety of heart or respiratory conditions. There are two types of ECMO: veno-venous (V-V) and veno-arterial (V-A) ECMO^(8)^.

The decision to use ECMO technology shall be shared among healthcare professionals and the provision of this technology shall be considered for patients with the possibility of improving the prognosis, according to the management protocols of the Acute Respiratory Distress Syndrome (ARDS)^(6)^. Thus, critically ill patients can benefit from being transported by aircraft while undergoing veno-venous or veno-arterial extracorporeal membrane oxygenation, so that they reach large centers safely and faster^(9)^.

In view of the above, the importance of managing the knowledge mastered by multidisciplinary teams, of operationalizing the guidelines for the care of critical patients with COVID-19, of implementing personal protective equipment and the processes for using them is highlighted. Moreover, training guided by science is relevant^(1)^. Thus, the dissemination of successful experiences that can contribute not only to the multiplication of information, but also to subsidize adjustments in the care behavior at services that experience similar situations, becomes appropriate.

In this case report, care for critically ill patients and the inherent difficulties of air transport with the patient on ECMO were described, with emphasis on the role of nurses in all phases of the process. Care management, work among high-performance multidisciplinary teams, and mastery of some particularities were also evidenced, with focus on quality care^(10–11)^.

Therefore, the objective of the study was to describe the experience of aeromedical interhospital transport of an adult patient with severe hypoxemic respiratory failure due to SARS-CoV-2, on extracorporeal membrane oxygenation.

## METHOD

### Design of Study

This is a case report, guided by the tool Case Report Guidelines (CARE), compliant with the CARE Checklist, with a descriptive approach. Case reports aim to expand global knowledge and have the potential to provide evidence for clinical research, to ensure quality improvement in practice and knowledge management^(12)^.

### Local

The present study took place in a private company that provides specialized services of aeromedical transport to users of private supplementary health insurance, and for public agencies, based in Belo Horizonte, in the State of Minas Gerais, Brazil.

### Selection Criteria

For this study, the first and only aeromedical interhospital transport of an adult patient with severe hypoxemic respiratory failure due to SARS-CoV-2, on extracorporeal membrane oxygenation, performed by the company above-mentioned, was selected.

### Data Collection

Data were collected from the patient’s digital medical record and the nurses’ field notes, two months after the transport was carried out, and from discussions among the multidisciplinary teams.

The flight lasted 1 hour and 25 minutes, the crew consisted of the pilot, co-pilot, on-board nurse, and the team of perfusionists/ECMO specialists, consisting of a nurse, a cardiologist, and a cardiovascular surgeon.

A collection instrument developed by the investigators was used, which consisted of the variables available in the medical record, related to the three phases of flight^(13)^.

In the first phase, called “Preflight”, there is the logistics, preparation of materials/equipment, assessment of laboratory tests, clinical conditions and aircraft equipment. In addition, the devices required for the care of critically ill patients were considered, such as: two mechanical ventilators, a portable gasometer, a portable ultrasound scanner, six infusion pumps, two multiparameter patient monitors with defibrillator, five portable oxygen cylinders, protection filters (HEPA/HME), and standardized bags containing kits for performing procedures.

In the second phase, the “in-flight” one, records, procedures to maintain stability, embarkation and disembarkation, equipment adjustments, intravenous infusions, and care for complications take place. In the third phase, called “post-flight”, cleaning, disinfection of equipment, organization and records in the company’s systems are carried out.

Image registration carried out by the professionals who worked in the transfer, in addition to being authorized, is available for free access on the Instagram social network profile of the company that performed the transport. Moreover, the assistance described was obtained through the digital medical record, the professional experience of the on-board nurses, the discussions based on scientific evidence that were guided by the management of knowledge among the multidisciplinary teams.

### Data Analysis

Data analysis was performed in a descriptive way from the information present in the digital medical record and the field notes.

### Ethical Aspects

The present case report followed the guidelines expressed in Resolution 466/12 of the National Health Council, which presents the ethical standards for conducting research with human beings and was approved by the Research Ethics Committee (CEP) of Faculdade Ciências Médicas de Minas Gerais, with CAAE no. 48361321.8.0000.5134 and opinion no. 4.831.047.

## RESULTS

### Case Report

On May 28, 2021, in the early evening, air medical interhospital transport was requested from a city located in the northeast of the capital of the State of Minas Gerais, about 450 km away. It was a male patient of 54 years weighing 100 kg, who was 1.85 cm tall, a sportsman, muscular, previously healthy and who was not on use of medication for preexisting diseases. There was a report of having used drug combinations at home to start early treatment for COVID-19, with medical indication (Azithromycin^®^, Hydroxychloroquine^®^ and Annita^®^ [Nitazoxanide]) and no improvement in symptoms.

The patient sought the emergency care of the hospital in his city of origin on May 19, 2021 and informed the physician on duty that the symptoms had started 17 days ago, with myalgia, fever, and fatigue. The patient was hospitalized with a diagnostic hypothesis of viral pneumonia and, on the following day, the test for the detection of COVID-19 (Reverse Transcription Polymerase Chain Reaction – RT PCR) was requested and confirmed the disease.

Chest computed tomography scans were performed, with an interval of three days. The first scan showed sparse ground- glass opacities with attenuation in both lungs, associated with thin interlobular septal thickenings in between, with less than 25% pulmonary involvement. In the second scan, still with a bilateral ground-glass pattern, a 50% lung involvement and a calcic nodule in the middle lobe were observed.

On May 24, 2021, corticotherapy associated with antibiotics was initiated. However, there was a gradual worsening of the clinical picture that progressed to respiratory failure. Referred to the Intensive Care Unit (ICU) of that hospital, the patient was intubated and the process of mechanical ventilation began.

Subsequently, the condition progressed to bilateral pneumothorax, diffuse subcutaneous emphysema, and bilateral chest drainage was performed. A chest computed tomography showed bilateral involvement, with ground glass pattern and 90% of lung involvement. There were three consecutive episodes of oxygen saturation drop during the two days prior to admission to the ICU, so that the minimum saturation found was 70%. In the meantime, the following invasive procedures were performed: femoral artery puncture, central venous access in the internal jugular, bladder catheterization, and nasoenteric catheterization.

On May 28, 2021, the assistant physician, in partnership with the family member from the medical area, discussed the case and came to the conclusion that the patient could benefit from the use of V-V ECMO. Thus, they contacted the ECMO reference center in the capital, which indicated the use of the technology.

Following experts’ agreement, the company’s operational management was contacted to initiate the procedures for interhospital aeromedical transport. The case was screened by the regulatory physician, who was emphatic about its complexity. He informed about the potential risks of transport, including the possibility of death during the transfer.

However, given the complexity, family members and assistant physicians still requested an on-site evaluation. Nevertheless, there were operational issues to be resolved, such as: limitation for night landing in the city of origin, the difficulty of getting a vacancy for COVID-19 ICU, and the situation that all ECMO technologies would be unavailable for use because they were being used in the capital city. Therefore, after great efforts to resolve operational issues, on the afternoon of May 29, 2021, interhospital aeromedical transport process began.

It should be mentioned that the workers were dressed for the care of COVID-19 suspected or confirmed cases. To this end, each member of the team wore a mask with a high protection factor against particles (N95), the hooded overalls to protect operations with biological risk and boots, face shield, cap, and overlapping surgical gloves.

After dressing, the on-board team (doctor and nurse) arrived at the hospital of origin for patient evaluation. It was found that the patient was in a serious condition, on mechanical ventilation, using sedative analgesics and neuromuscular blockers. However, the patient was hemodynamically unstable, on vasoactive drugs and borderline mean blood pressure. Serial blood gas analyses were performed, followed by the endotracheal aspiration procedure using a closed system. During the procedure, the patient reached worrying saturation levels (52%) and the procedure had to be interrupted due to ventilatory instability.

For that reason, the on-board team, the assistant physician, the family member of the medical area discussed the case, and reached a consensus that, at that moment, transport would not be possible, as the risk outweighed the benefits. Therefore, the multidisciplinary team returned to the operational base with an empty aircraft, in the late afternoon of May 29, 2021. Nevertheless, the teams maintained effective communication so that they could carry out the interhospital aeromedical transport at the best possible time and safely.

On May 31, 2021, the teams agreed on an action plan among the ECMO specialists, the origin hospital’s intensivists, the flight team, and operational management.

The first stage was carried out by the team of perfusionists/ECMO specialists, which consisted of a nurse, a cardiologist, and a cardiovascular surgeon. The day before the air transport, the team of perfusionists traveled in an executive aircraft to assess and stabilize the patient. Thus, they took all the technology to start ECMO with them: the complete extracorporeal oxygenation system (centrifugal pump, oxygenator, circuits, cannulas, and the oxygenator). After the team arrived at the requesting hospital, the ECMO team surgeon performed bedside implantation of the femorojugular cannulas (F>J) to start using the technology, with the aim of improving clinical conditions for transport.

The second stage was carried out and based on an agreement among the ECMO team, operational management, and the flight team, who was in the capital city. The aerial ICU, fixed- wing aircraft – King Air-B200, after approval by the ECMO team, moved to the city of origin with the crew and the on-board intensive care nurse, responsible for preparing all materials and equipment for the preflight phase.

**Chart 1.**
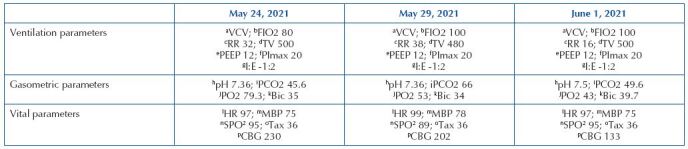
Ventilatory, blood gas and vital parameters, in chronological order, used to assess interhospital aeromedical transport – Belo Horizonte, MG, Brazil, 2021.

^a^Volume-cycled ventilation; ^b^Fraction of inspired oxygen; ^c^Respiratory rate; ^d^Tidal volume; ^e^Positive pressure at the end of expiration; ^f^Maximum inspiratory pressure; ^g^Inspiratory:expiratory ratio; ^h^Hydrogen potential; ^i^Partial pressure of carbon dioxide; ^j^Partial pressure of oxygen; ^k^Serum bicarbonate; ^l^Heart rate; ^m^Mean blood pressure; ^n^Peripheral oxygen saturation; ^o^Axillary temperature; ^p^Capillary blood glucose.

On June 1, 2021, the on-board nurse verified, upon arriving at the hospital of origin, that the patient was on the 21st day after positive test for COVID-19, with severe hypoxemic respiratory failure due to SARS-CoV-2, hypoxemia refractory to clinical measures, P/F ratio of 60-80 for 48 hours, and was deeply sedated (Ramsay scale score 6). He was intubated, device No. 8.0, fixation 22.0 cm; cuff pressure maintained at 23 mmHg; continuous ETCO_2_ reading at 26 mm/Hg. Ventilatory parameters remained high and chest X-ray showed bilateral involvement and diffuse infiltrate. The patient had been cannulated for 24 hours for V-V ECMO (Flow 5.0; FIO2 100% and SWEEP 6.0) with improvement of general medical conditions and of blood gas data. The data of the parameters analyzed are presented in Chart 1.

The results of laboratory tests on June 1, 2021 were: global leukocyte count, 12,900 (mm^3^); red blood cells, 9.3 (million/mm^3^); hematocrit, 27 (%); platelets, 181,000 (mm^3^); urea, 82 (mg/dL); creatinine, 1.4 (mg/dL); sodium, 150 (mEq/L); potassium, 3.8 (mEq/L); calcium, 1.19 (mg/dl); and venous lactate, 13.1 (mmol/L).

Prior to the transfer, the patient remained in a very serious clinical condition, on continuous use of sedative analgesics, neuromuscular blockers, vasoactive drugs, heparin titrated by Activated Partial Thromboplastin Time, of 4/4 hours. In addition, blood cultures, urine cultures and tracheal secretion cultures were collected for 24 hours, and correction of hypernatremia was performed with 0.45% sodium chloride solution.

The guidelines for interhospital aeromedical transport were followed to optimize safety and quality of care. Following general assessment, preparations for the “pre-flight” phase of transport by the on-board nurse were initiated: change of devices for continuous drug infusion (neuromuscular blockers; sedative analgesics; heparin and noradrenaline); circuit replacement and adaptation of the transport ventilator; performance of arterial blood gas analysis; adjusment and fixation of the V-V ECMO system (adapted to a base and a tripod over the patient); change of invasive and non-invasive monitoring to the multiparameter monitor; emptying of the urine closed system; patient transfer from hospital bed to aircraft transport stretcher – Lifeport^®^. Seat belts were adjusted, fastened, and all equipment was secured.

After the actions described, the team conducted the patient to the airport from the city of origin with support from the land ambulance, which totaled ground time of 03 h and 25 min, with no complications. The crew team consisted of two specialist nurses (intensivist/on-board; perfusionist), two physicians (cardiovascular surgeon and intensivist cardiologist/perfusionist) and two crew members (pilot and copilot).

During the in-flight phase, which totaled 01 h and 25 minutes, there were no technical complications or instability, despite the patient’s severity. Methodical care was based on scientific evidence, which was individualized, aligned with advanced practices, and probably contributed to the positive transport outcome. To this end, at this stage, the main objective was to keep the patient’s stability, ensure safety and quality of care.

Therefore, the procedures common to the multiprofessional team and necessary for this stage were carried out, such as: paying attention to the team’s attire; changing the oxygen delivery system; monitoring the cabin temperature; connecting the equipment to the Lifeport^®^ base’s electrical grid; adjusting the equipment fixtures; allocating the bags with reserve materials; controlling the ECMO system, and ensuring cannulas patency.

**Figure 1.**
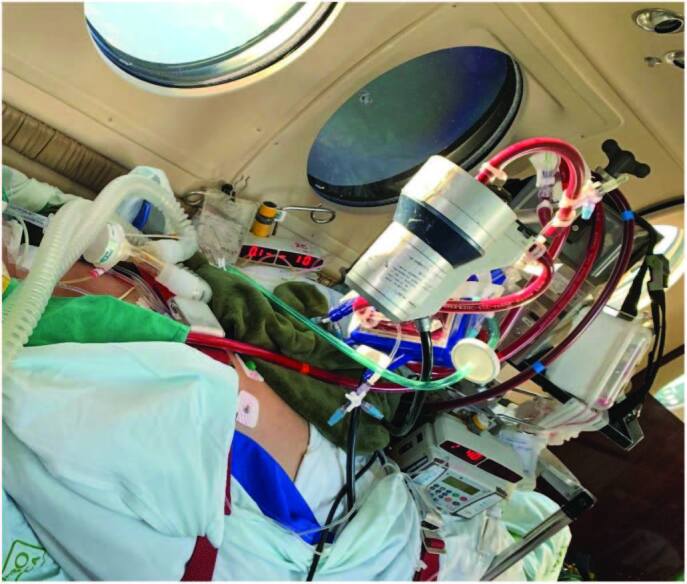
Interhospital air transport of an adult with COVID-19 using ECMO, in the “in-flight” phase.

The on-board nurse, in particular, supervised all the procedures mentioned above; in addition, he was responsible for preparing and controlling the infusion of continuous medications through infusion pumps; performing drug adjustments according to medical requests; positioning the bed head; measuring pressure of the endotracheal tube cuff; monitoring the capnography curve; aspirating endotracheal tube through a closed system; opening the closed system of the indwelling urinary catheter; recording vital parameters on the electronic flight record; performing blood gas analysis and laboratory tests; maintaining the patent invasive monitoring system, performing the water balance, among others (Figure 1).

It is worth remembering that the phases of embarkation and disembarkation of the critical patient on ECMO, in the aircraft and in the ambulances, required extreme attention from the multiprofessional team. In both vehicles or phases, the patient was slid onto the bases of the Lifeport^®^ stretcher with the help of the entire team, who moved in syncrony.

It should be noted that the simultaneous handling of the patient by the team, along with the use of equipment, limited internal space of the aircraft and narrow door, was difficult and demanded patience and effective communication among the team. Finally, the procedure required four people on the ground to receive the patient safely. The dynamics of embarkation and disembarkation were rigorous, requiring skill, experience, and attention to the smallest details. The teams had to be synchronized, and communication in closed loop, continuous evaluation of processes, care with materials and equipment to avoid loss of devices and/or adverse events were required.

When landing at Pampulha Airport, an advanced ground support ambulance was waiting with a multidisciplinary team, but due to the high complexity of transport, the patient was kept on the Lifeport^®^ aircraft stretcher, to minimize the risks of transfer, prioritize the quality of care, and focus on patient safety. The on-board nurse accompanied the multiprofessional advanced ground support ambulance team to finalize the transport, transferred the case to the nurse at the destination hospital, and collected the delivery record from the final physician in charge. Subsequently, he returned to the operational base with all the materials and equipment, to carry out the guidelines for the final stage.

In the post-flight phase, the on-board nurse was responsible for organizing, cleaning, disinfecting all materials and equipment used. After these procedures, the equipment was reconnected to the electrical grid, the kits were relocated, the expense sheet filled in, records were made in the report book and in the company’s information systems. It should be noted that, with the pandemic, the cleaning and disinfection processes were optimized, with the use of the sprayer for high-level disinfection.

Finally, transport complexity, due to its uniqueness, demanded advanced practices of multiprofessional articulation and aimed at qualified assistance, and the air medical transport of the patient on ECMO was a relevant opportunity for the teams to share and assimilate new knowledge. Despite all challenges, health services were successful in delivering the critically ill patient in a stable condition, considering the level of severity. It should be noted that 45 days after V-V ECMO, the patient was decannulated and subsequently discharged from the ICU.

## DISCUSSION

In Brazil, there is a knowledge gap regarding the role of nurses in the areas of aerospace medicine and perfusionism. For this reason, this case report becomes important for the dissemination of the theme.

In this scenario, the on-board nurse’s praxis is protected by Law No. 7.498/96, which regulates Nursing Professional Practice, establishes that it is the nurses’ exclusive role to organize, direct care for critical patients, and perform highly complex activities. In addition, COFEN Resolution 0551/17, which regulates the role of nurses in Pre-Hospital Mobile care and Interhospital Care in Air Vehicles, cites the need for professional specialization or of being already working in the area until the publication of the resolution^(14)^.

It should be noted that the results of the present report indicated that the multidisciplinary team, after discussing the case, concluded that the patient could benefit from the use of ECMO. Therefore, the procedures for transfer to the center accredited by ELSO were initiated. Two North American studies show different results. The first emphasizes a positive clinical outcome with the use of ECMO as an adjuvant, according to the case report of the transport of patients diagnosed with COVID 19 and on ECMO^(1)^. In contrast, the second points out that the use of this technology has high mortality rates, discusses the multifactorial aspects of ECMO and the need for transfer to accredited centers^(15)^. In view of the above, it is observed that the use of ECMO is controversial and requires further research, so it is presented as a therapeutic option whose clinical decision on its use requires singular planning, a multiprofessional approach, and risk-benefit assessment.

It was evident that the indication of ECMO, as well as the need to reduce travel time and the distance from the city of origin to the ELSO accredited center, were factors that made fixed-wing air transport the preferred strategy for transfer, with safety and quality. This fact is confirmed by North American researchers who claim that the types of aircraft used influence the duration of air transport, associated logistics, physiological changes in altitude and, consequently, can collaborate so that definitive assistance is received in a timely manner^(16)^.

Research carried out in the United States pointed out that the air transport of patients on ECMO, with high-fidelity simulation, added to the infection by the Coronavirus, increases the complexity of air transport^(1)^. This fact corroborates the Arab case report of a child with a single ventricle, and severe myocardial dysfunction, with an indication for heart transplantation using ECMO, who was transported from Qatar to Belgium. The authors described the transport in fixed-wing aircraft as more complex, as it requires ground ambulance teams at the airports of origin and destination, for the transfer between hospitals, much electrical equipment, optimized oxygen supply, and temperature control^(17)^.

Therefore, the multidisciplinary team has to be specialized, and must master the care processes inherent to the air environment and, consequently, have the ability to handle the technologies associated with the hypobaric environment.

Due to the difficulties reported in embarkation and disembarkation, amplified with the use of ECMO, the limitation of space in air and land ambulances and the dynamics of patient movement, it is pointed out that the risk of adverse events is increased in the operation of this type of transport. Series of cases from the Mayo Clinic, in the United States, pointed out that, during embarkation and disembarkation of five critical patients using ECMO, both in the aircraft and in ground support ambulances, professionals faced several factors such as limited spaces, the need for continuous care, communication problems, high number of equipment that required an electrical grid and high consumption of portable oxygen^(18)^.

These findings are in line with research carried out in Stockholm on adverse events during transport using ECMO, whose results showed that the offer of the technology is not only centralized in high-complexity hospitals due to the high cost, and the need for highly specialized teams, but also demands the existence of an experienced operational management^(8)^. Thus, the relevance of training on the air transport of patients on ECMO to contemplate the particularities of the embarkation and disembarkation process is highlighted.

It was evidenced that the multifactorial interfaces present in all flight phases can make health care processes difficult. However, the transport was carried out by qualified teams, with aligned logistics, well-established processes and, consequently, this contributed to the prevention of potential risks and possible adverse events. In what it refers to transport complexity, the report exemplifies the need to master advanced practices, which aim at qualified assistance and, for that, knowledge management and investment in training are necessary.

A North American, retrospective study, guided by the ELSO guidelines for the care of patients with COVID 19 on ECMO, evaluated the aeromedical helicopter program of the reference center and showed that, to work in such a service, the multiprofessional teams needed to develop the capacity to work together, through knowledge management about the flight phases^(19)^.

Regarding the qualification of human resources to work in aeromedical transport, a Canadian qualitative study carried out with leaders from different continents, highlighted the strategies for the translation of knowledge based on multiprofessional interaction and praxis aligned with science as promising^(20)^. In addition, Brazilian research, with a qualitative approach, observed that the constructions of everyday teamwork in aerospace medicine are permeated by joint, synchronous, and collaborative action among doctors and nurses^(21)^. Thus, it is pertinent that the multiprofessional relationship of the aerospace health team be the target of research, for the construction of scientific evidence that guides the practice in this scenario requiring such specificity.

Therefore, the need for effective training of teams through multiprofessional interaction, investment in research on ECMO and its use in aircraft are relevant to contribute to the state of the art of the subject, direct the Evidence-Based Practice and, therefore, optimize quality of care and patient safety. Therefore, it should be emphasized that the mastering of processes with the use of new tools and the dissemination of studies on these processes can contribute to the promotion of new knowledge^(20)^.

It is also noteworthy that the result of this report showed the leading role of nurses in all flight phases, until the successful transfer of the patient to the ELSO-accredited hospital, which shows the importance of this professional, not only for the feasibility of the transport, but to increase the quality and effectiveness of health care in the airspace context. This corroborates the experience report of Brazilian nurses, in what regards the structuring and management process for the care of patients with COVID 19, which showed protagonism of nurses in the pandemic, because due to the managerial, educational and care attributions, inherent to the nursing professional practice, they show multifaceted performance and expertise of critical patient care, care planning, the construction of protocols and the provision of direct care^(11)^. Therefore, the consolidation of the nurse as a relevant member of the multidisciplinary health care team is observed.

In view of the above, for the entire process of air transfer of the patient on ECMO to be carried out with safety and quality, there was a sharing of responsibilities, knowledge and, consequently, the possibility of synchronous and successful multidisciplinary work.

The scarcity of publications on the air transport of patients on ECMO in Brazil stands out as the main limitation of the study, especially in the nursing area. Therefore, this case report can help on-board nurses to support scientific evidence-based practices, guided by the flight phases, especially in knowledge management and in the leading role of specialist nurses.

## CONCLUSION

The success of interhospital aeromedical transport of the adult patient with severe hypoxemic respiratory failure due to SARS-CoV-2 using extracorporeal membrane oxygenation was observed, and after 45 days he was decannulated and discharged from the ICU.

Such a successful experience is relevant since critical patients, with respiratory failure and infected by SARS-CoV-2, on ECMO, can benefit from transport in fixed-wing aircraft when they are transferred quickly and safely to hospitals in large centers, by highly specialized multiprofessional teams. Above all, the relevant role of on-board nurses, who conducted all phases of the flight, managed the processes, and overcame the difficulties inherent to transport in a hypobaric environment, shall be highlighted.

## ASSOCIATE EDITOR

Thereza Maria Magalhães Moreira
